# Lifestyle changes among older adults during and after COVID-19 pandemic in Lithuania

**DOI:** 10.3389/fpubh.2024.1504193

**Published:** 2025-01-06

**Authors:** Skirmante Sauliune, Ramune Kalediene, Vytenis Kalibatas, Snieguole Kaseliene, Olga Mesceriakova

**Affiliations:** Department of Health Management, Faculty of Public Health, Lithuanian University of Health Sciences, Kaunas, Lithuania

**Keywords:** COVID-19, pandemic, lifestyle, eating habits, physical activity, social engagement, smoking, alcohol consumption

## Abstract

**The aim of this study:**

to analyze lifestyle changes among older adults during and after COVID-19 pandemic in Lithuania, with a particular focus on eating habits, physical activity, social engagement and harmful habits.

**Methods:**

The representative sample of Lithuanian population over 65 years old (1,503 individuals) was involved in the questionnaire survey, performed in January 2024.

**Results:**

Most of the eating habits and the body weight of the older adults did not change during the COVID-19 pandemic in Lithuania. Respondents noted that their physical activity decreased, face-to-face communication became rarer, while remote communication increased. During pandemic, the increased frequency of snacking was more often indicated by persons with lower than secondary education, working, receiving higher incomes, as well as increased snacking and body weight were more prevalent among younger, single or overweight persons; increased body weight during the pandemic was more often mentioned by the persons who assessed their health poorly. Physical activity decreased more often among rural residents and groups with higher or college education; face-to-face communication has become rarer among younger people, rural residents, pensioners, disabled, people with higher income and those with overweight, while remote communication has increased among women, respondents with a university education and those with an average income (*p* < 0.05). Nearly half of the respondents indicated that the changes in food consumption and smoking frequency that occurred during the pandemic remained after it. After the pandemic, the habits of consuming fast food, confectionery or other sweets and changes in body weight mostly remained, while the patterns of communication returned to the pre-pandemic level. Most of the healthy lifestyle habits formed during the pandemic persisted after the pandemic. The lifestyle habits formed during the pandemic remained more stable in the groups of older persons, residents of smaller towns, respondents with lower than secondary education, higher income, singles, disabled, obese, and those who assessed their health poorly (*p* < 0.05).

**Conclusion:**

During the COVID-19 pandemic in Lithuania, there were various changes in eating habits, physical activity, communication patterns, and harmful habits. Certain habits formed during the pandemic continued afterward, particularly among specific groups of the older Lithuanian adults.

## 1 Introduction

The COVID-19 pandemic has had, and continues to have, various consequences for the health and lifestyle of people around the world, especially for the older adults. This unprecedented public health crisis necessitated widespread lifestyle changes due to the imposition of social distancing measures, lockdowns, and other preventive strategies aimed at mitigating virus transmission (1). Consequently, these measures have influenced the daily routines, physical activities, social interactions, and overall well-being of older adults, leading to significant lifestyle modifications during and after the pandemic (2–6).

Analyzing lifestyle, 65 aged and older people appeared to be among the most vulnerable groups during the pandemic and were given special attention. Social isolation during the COVID-19 pandemic has had a significant impact on lifestyle, especially sedentary lifestyle and resulting reduced physical activity, sleep and eating habits. Older adults were often unable to adapt to restraint/quarantine measures and suffered from depression and cognitive impairment (7). Spending more time at home was associated with eating more and sitting longer. The stress associated with fear and the constant media coverage of the pandemic has led to the consumption of so-called “convenience foods” (high in added sugar or fat) or increased snacking between meals, as well as alcohol consumption (8).

The COVID-19 pandemic significantly impacted alcohol consumption and smoking habits. In Germany, 35.5% of respondents reported increased drinking during quarantine, while 45.8% reported increased smoking, often due to heightened stress (9). In Italy, 9.1% of respondents reported increased cigarette consumption, with 9.0% starting, relapsing, or intensifying their smoking (10). In South Korea, 11.0% of current smokers increased smoking, whereas 12.8% decreased their smoking during the pandemic (11).

The aftermath of the pandemic presents a unique opportunity to examine the long-term effects of these lifestyle changes. Unfortunately, there are not many country-wide studies analyzing the lifestyle changes of the population aged 65 and older during the COVID-19 pandemic, they are fragmentary, often only incorporated in the larger studies. It is crucial to understand how the temporary adjustments made during the pandemic might influence the future behavior and health outcomes of older adults, especially for countries with an aging population. Lithuania is one of the countries with rapidly aging population, with a fifth of the population aged 65 and over (12). The aging population creates challenges both for health and social care services as well as older adults are characterized by different lifestyle and health problems compared to other age groups. Therefore, it is extremely important to analyze the current situation, respond to it, intervene early, and prevent further consequences of the pandemic. Therefore, the aim of this study was to analyze lifestyle changes among older adults during and after COVID-19 pandemic in Lithuania, with a particular focus on eating habits, physical activity, social engagement and harmful habits.

## 2 Materials and methods

### 2.1 Research organization

The study was conducted in January 5–17, 2024, in Lithuania. The study was conducted in January 5–17, 2024, in Lithuania. 1,503 Lithuanian residents 65 years of age and older were interviewed by means of an anonymous survey (the response rate was 36%; Table 1). The research was conducted in the form of face-to-face interviews at the respondent’s home by an independent institution of public opinion and market research “Vilmorus Ltd.” A team of professional interviewers collected the data. The duration of one interview was 25–40 min. The survey covered 34 municipalities - it took place in 26 cities and over 50 villages. All the participants were informed about the study. Participation was voluntary and anonymous. Verbal consent to participate in the study was received from all the participants.

**Table 1:** Peculiarities of conducting the survey.

Selected contacts	*n*	%
Participated in the survey	1,503	36.0
Refused to participate	432	10.4
No one was at home	918	22.0
Ineligible due to age/gender	962	23.0
Other (locked stairwell, angry dog, respondent drunk, impossible to communicate, dangerous to enter, uninhabited house, etc.)	360	8.6
Total contacts	4,175	100

1,503 Lithuanian residents 65 years of age and older were interviewed by means of an anonymous survey (the response rate was 36%; Table 1) by the application of face-to-face interviews at the respondent’s home. The duration of one interview was 25–40 min. The survey covered 34 municipalities—it took place in 26 cities and over 50 villages.

### 2.2 Sampling

The study population of the survey is residents of Lithuanian households aged 65 and older selected using a multi-stage, probabilistic and proportional sampling technique. This method ensured equal opportunities for all Lithuanian residents, representing different places of residence and demographic groups, to participate in the study. The sample had to correspond to the demographic distribution of the Lithuanian population in terms gender and place of residence. The sample of Lithuanian residents older than 65 years was compiled based on the address register of the Republic of Lithuania managed by the state company Centre of Registers. A “route method” was used for further household selection. The survey was conducted in all ten administrative regions (counties) of Lithuania. The number of individuals interviewed in each county was proportional to its population size, as established using national statistical data. Within each county, the county center or another town and rural localities were chosen through random selection. Next, streets and the starting point of the route were randomly selected in each location. To ensure a representative respondent within each household, the ‘youngest male rule’ was applied. If the intended respondent was unavailable, a subsequent visit was arranged. Open Epi sample size calculator was used for the estimation of sample size using the following parameters: population size for each, gender and place of residence group, anticipated frequency 50%, probability of the mistake 5%, design effect 1 (estimated sample size was 1,536 participants).

### 2.3 Contingent

The distribution and grouping of respondents according to socio-demographic characteristics is presented in Table 2. Lithuanian residents aged 65–96 years participated in the study. The median age was 73 years, mean—74.04 years (standard deviation 6.37 years). The distribution of the research participants by age, gender and place of residence did not differ significantly from that of the whole population of Lithuania, so the study sample can be considered as a representative for the population of Lithuania.

**Table 2:** Distribution of respondents according to socio-demographic characteristics.

Factors	Ungrouped responses	*n*	%	Grouped responses	*n*	%
Gender	Males Females	528 975	35.1 64.9	–	–	–
Age (years)	65–69 70–74 75–79 80–84 85–96	437 376 392 197 101	29.1 25.0 26.1 13.1 6.7	65–74 75–84 85 and older	813 589 101	54.1 39.8 6.7
Education	Elementary or lower Lower secondary / unfinished secondary / vocational without secondary Secondary Professional with secondary Higher (and technical) College University Did not indicate	42 137 332 296 388 27 278 3	2.8 9.1 22.1 19.7 25.8 1.8 18.5 0.2	Lower than secondary Secondary (secondary + professional with secondary) Higher and college (higher / technical + college) University	179 628 415 278	11.9 41.9 27.7 18.6
Main occupation	Pensioner Disabled Employed Employed pensioner Disabled pensioner	1,309 27 71 68 29	87.1 1.8 4.7 4.5 1.9	Pensioner Disabled (disabled + disabled pensioner) Employed (employed + employed pensioner)	1,309 56 138	87.1 3.7 9.2
Number of persons in the household	1 2 3 4 5 6	617 746 96 33 10 2	41.0 49.6 6.4 2.2 0.6 0.1	1 2 and more	617 886	41.0 59.0
Marital status	Single Married Live together without marriage Divorced Widowed	48 670 65 161 559	3.2 44.6 4.3 10.7 37.2	Married Single (single + divorced + widowed)	670 768	46.6 53.4
Income (after the taxes for each family member per month)	250 EUR or less 251–500 EUR 501–750 EUR 751–1,000 EUR More than 1,000 EUR Did not indicate	22 596 621 197 54 12	1.5 39.7 41.3 13.1 3.6 0.8	500 EUR or less 501–750 EUR More than 750 EUR	618 621 251	41.5 41.7 16.8
Place of residence	Vilnius Kaunas, Klaipėda, Šiauliai, Panevėžys Other towns Village	254 350 399 500	16.9 23.3 26.6 33.2	City Town Village	604 399 500	40.2 26.6 33.2

The distribution of respondents according to certain health-related characteristics - body mass index (BMI), subjectively assessed health status was also rated. Study participants were also asked if they had a history of COVID-19. The distribution and grouping of respondents according to these health-related characteristics is presented in Table 3.

**Table 3:** Distribution of respondents according to health characteristics.

Factors	Ungrouped responses	*n*	%	Grouped responses	*n*	%
BMI	Too low (under 18.5) Normal (18.5–24.99) Overweight (25–29.99) Obesity (30 and over) Did not specify height and / or weight	6 427 693 363 14	0.4 28.4 46.1 24.2 0.9	Too low / normal (under 25) Overweight (25–29.99) Obesity (30 and over)	433 693 363	29.1 46.5 24.4
Subjectively assessed health	Good Pretty good Average Pretty poor Poor	64 206 888 246 98	4.3 13.7 59.1 16.4 6,5	Good / pretty good Average Pretty poor/ poor	270 888 344	18.0 59.1 22.9
Have you had the disease COVID-19?	Yes, confirmed by the test Yes, not confirmed by the test I wasn’t sick I do not know	593 167 638 105	39.5 11.1 42.4 7.0	Yes, confirmed by the test I wasn’t sick I do not know (do not know + yes, not confirmed by test)	593 638 272	39.5 42.4 18.1

### 2.4 Research instrument

An original questionnaire of 74 questions was prepared for the study. The questionnaire consisted of 3 thematic sections (general information, lifestyle and its changes during and after the COVID-19 pandemic, and access to healthcare during and after the COVID-19 pandemic). This article analyses questions related to lifestyle changes during the COVID-19 pandemic (7 questions about changes in food consumption and 8 questions about changes in other lifestyle factors); lifestyle changes after the end of the pandemic (7 questions about changes in food consumption and 8 questions about changes in other lifestyle factors).

### 2.5 Statistical analysis

Statistical data analysis was performed using the IBM SPSS Statistics 29 statistical package. Data was weighted by gender and age. To identify the most vulnerable older adults population groups, study participants’ answers to questionnaire questions were compared according to socio-demographic factors and health characteristics using the Chi-square (χ^2^) criterion, and the z criterion for pairwise comparison of frequencies. Associations were considered statistically significant when the probability of the mistake (p) was <0.05.

## 3 Results

### 3.1 Lifestyle changes during a COVID-19 pandemic

**Figure 1:**
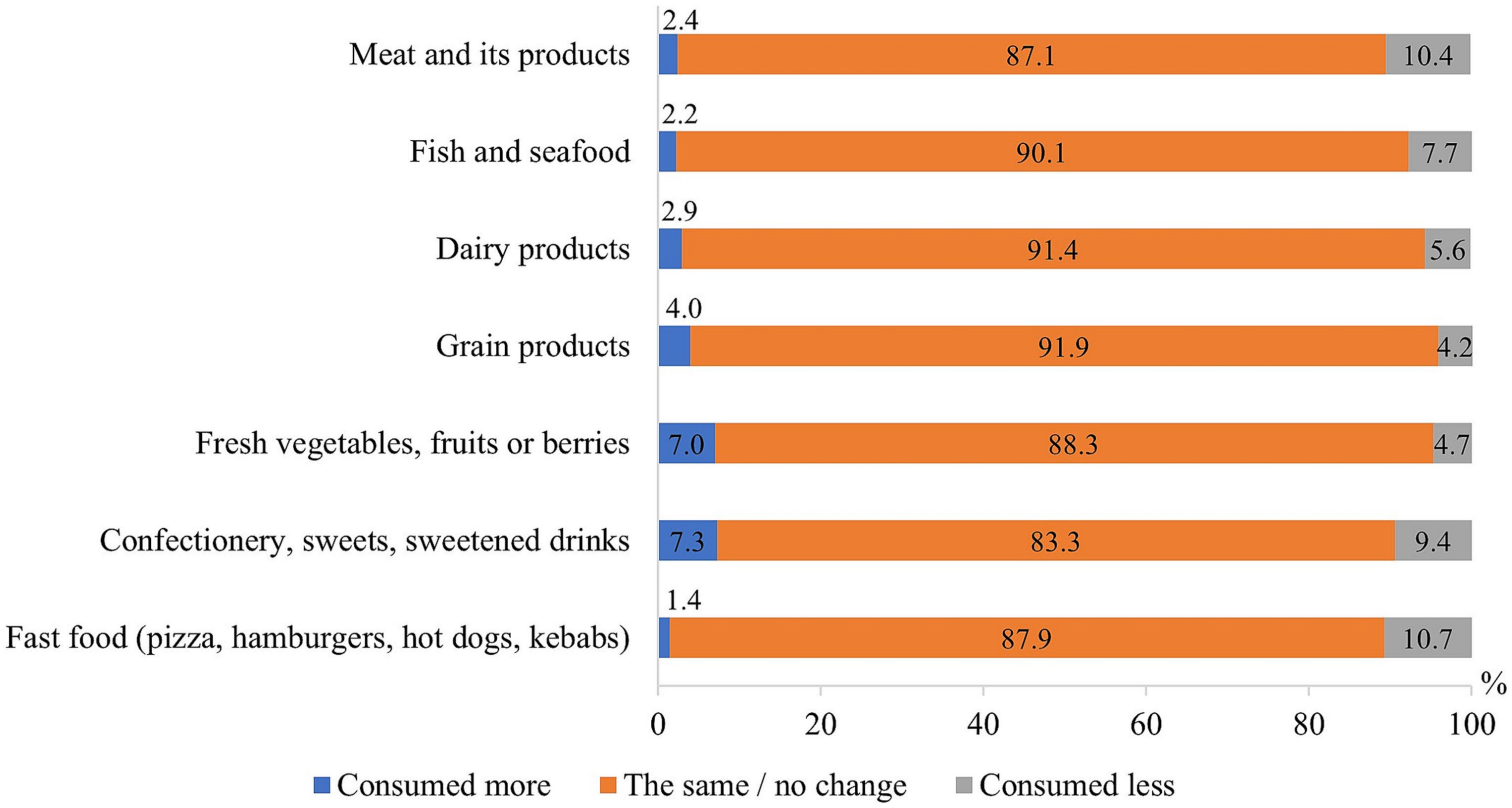
Changes of eating habits during the COVID-19 pandemic.

Most of the respondents indicated that their eating habits did not change during the COVID-19 pandemic, and only 7.3% mentioned that they consumed more confectionery or other sweets, 7.0%—fresh vegetables, fruits or berries, every tenth study participant consumed less fast food (10.7%), meat and its products (10.4%) and confectionery or other sweets (9.4%), (Figure 1).

**Figure 2:**
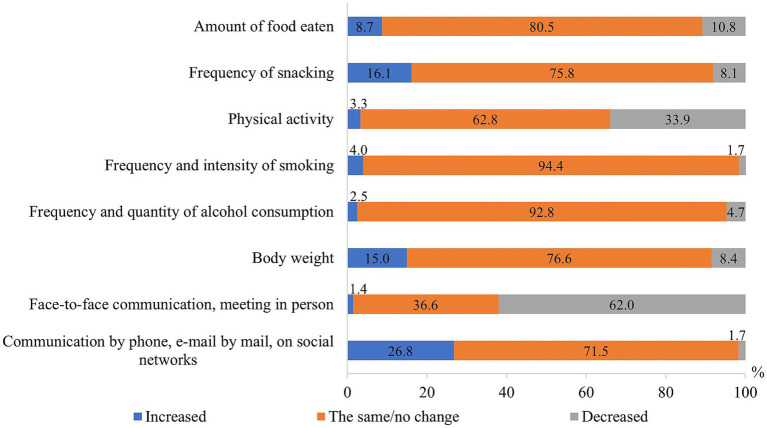
Changes of some of the lifestyle factors during the COVID-19 pandemic.

Also, most respondents indicated that the frequency and intensity of their smoking (94.4%), the frequency and amount of alcohol consumption (92.8%), the amount of food eaten (80.5%), snacking frequency (75.8%), and body mass (76.6%) did not change during the COVID-19 pandemic. However, a considerable number of study participants noted that their communication with their relatives “live” by meeting has become rare (62.0%) and distance communication has increased (i.e., by phone, e-mail or social networks; 26.8%), 33.9% indicated that their physical activity has decreased, 16.1% - snacking has increased, and 15% observed an increased body mass (Figure 2).

**Figure 3:**
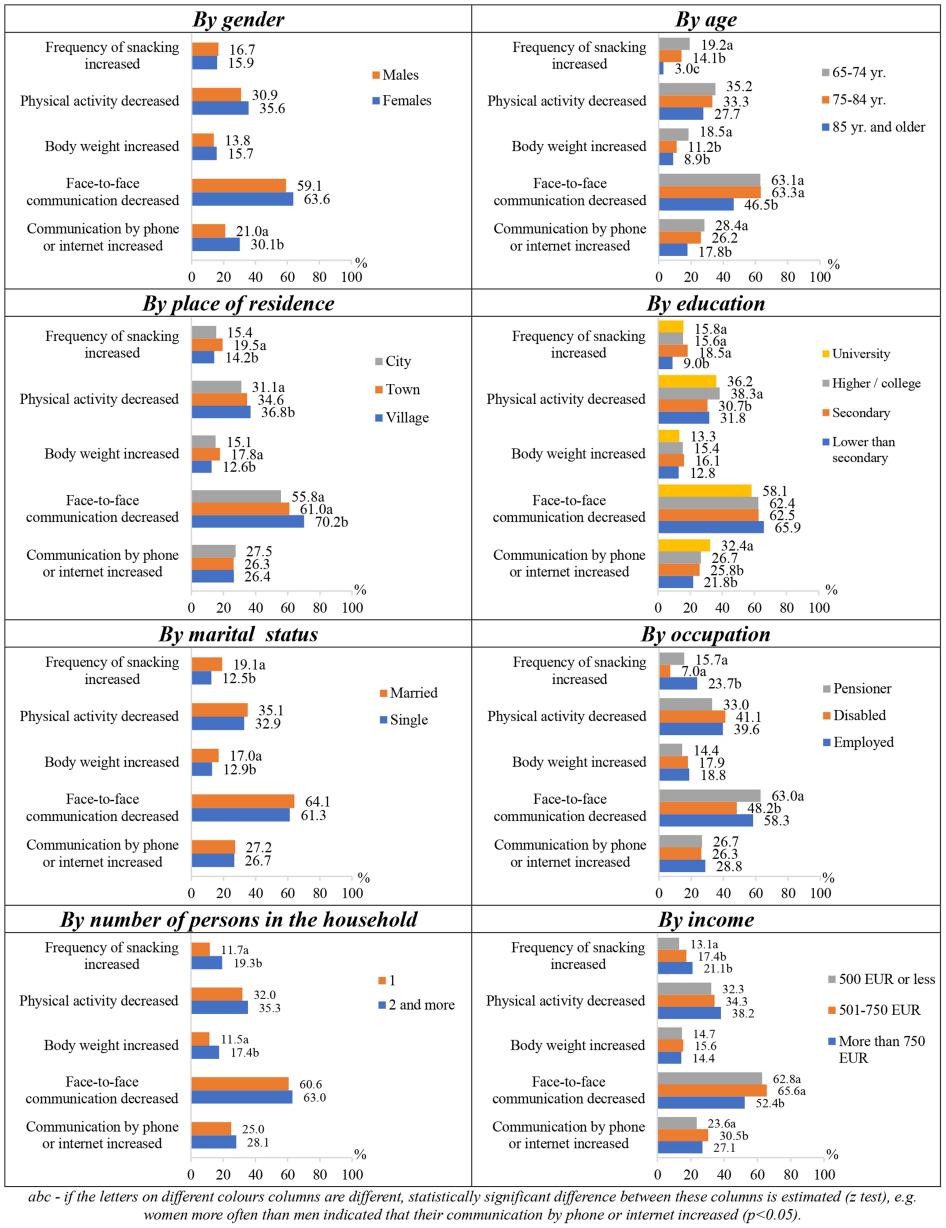
Changes in lifestyle factors during the COVID-19 pandemic depending on socio-demographic factors.

Changes in respondents’ lifestyle factors during the COVID-19 pandemic were statistically significantly (*p* < 0.05) associated with their sociodemographic characteristics (Figure 3). Women more often than men indicated that their distance/online communication improved. Younger respondents more often indicated more frequent snacking and an increase in body weight compared to older ones. They also reported more frequent communication with relatives at a distance and less frequent communication by “in person” meetings. Those living in the countryside more often than the inhabitants of the cities noted that their physical activity decreased and communication during meetings became less frequent, while the inhabitants of smaller towns more often than those living in the countryside stated that their snacking frequency and body weight increased. Respondents with lower than secondary education more seldom reported that their frequency of snacking had increased compared to the respondents of other educational groups. Physical activity of those with higher or college education more often decreased in comparison to those with secondary education. Respondents in the university education group more often than persons with lower education reported increased communication by phone or the Internet. Married respondents or those living in the household with other person, more often noticed that their snacking frequency and body weight increased as compared to the single ones. In the group of working persons, snacking became more frequent than among others, and pensioners more often than disabled persons complained that their communication with relatives “live meeting,” became less frequent. As the income of the respondents increased, the frequency of their snacking also increased; those with an income of EUR 750 or less more often than those with a higher income indicated that their communication with relatives by meeting became rare, and those whose income reached EUR 501–750 more often than those with a lower income indicated that their communication over a distance has increased.

Overweight respondents more often stated that their frequency of snacking increased, in comparison to those with too low/normal weight respondents (18.0% vs. 13.4%); they also more often stated that their “live” communication, when meeting, became less frequent compared to low/normal weight and obese respondents (66.4% vs. 60.3% vs. 55.8%). The higher the respondents’ BMI was, the more often they reported that their body weight increased during the pandemic. According to the subjectively assessed health status, respondents who rated their health poorly complained of increased body weight the most compared to those who assessed their health average and pretty good/good (20.9% vs. 13.5% vs. 12.2%).

### 3.2 Lifestyle changes after the COVID-19 pandemic

**Figure 4:**
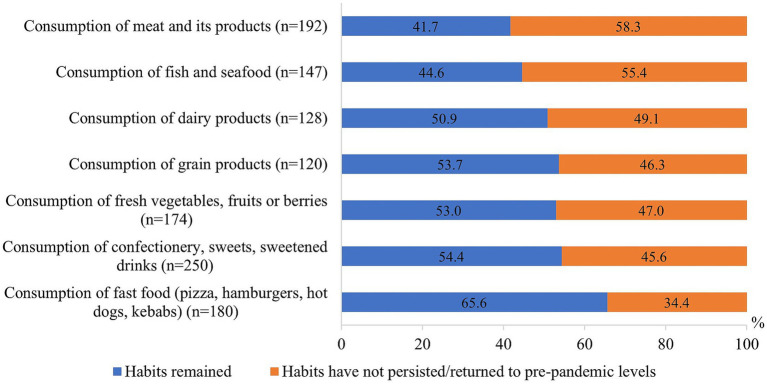
Changes of eating habits after the COVID-19 pandemic.

Nearly half of the respondents reported that the changes in food consumption that occurred during the COVID-19 pandemic remained, while the other half said that their food consumption returned to the pre-pandemic level (Figure 4). The largest number of respondents indicated that their habits of consuming fast food (65.6%) and confectionery and other sweets (54.4%) remained, while meat and its products (41.7%) as well as fish and seafood remained the least common (44.6%).

**Figure 5:**
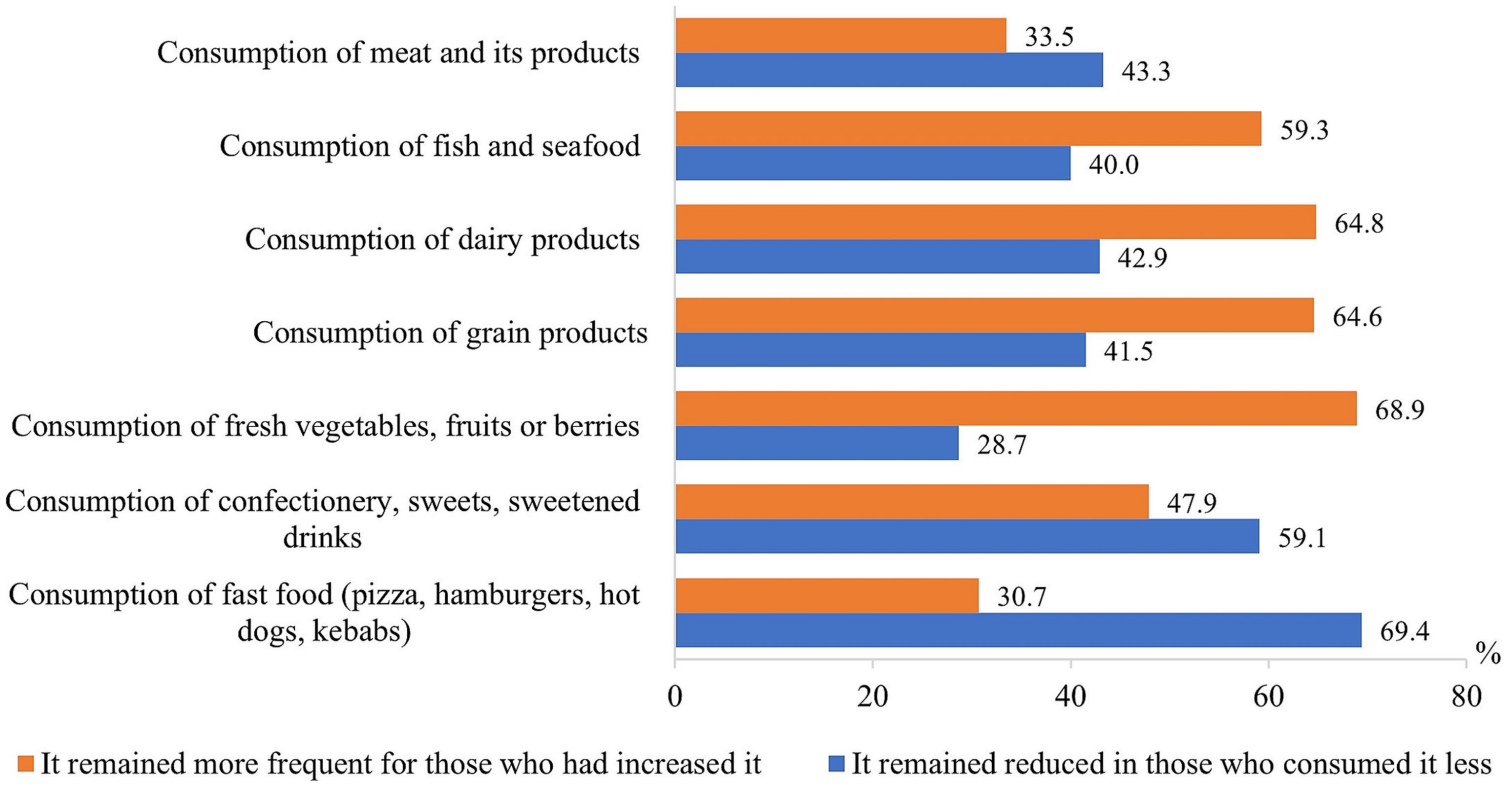
Proportion of respondents who maintained changes formed during the pandemic in eating habits after the end of the COVID-19 pandemic.

The consumption of fish and seafood, dairy and grain products, fresh vegetables, fruits and berries remained more frequent after the pandemic among those who had increased it during the pandemic, while the consumption of meat and its products, confectionery and other sweets, and fast food remained more frequent for those who used them less during the pandemic than before it (Figure 5).

**Figure 6:**
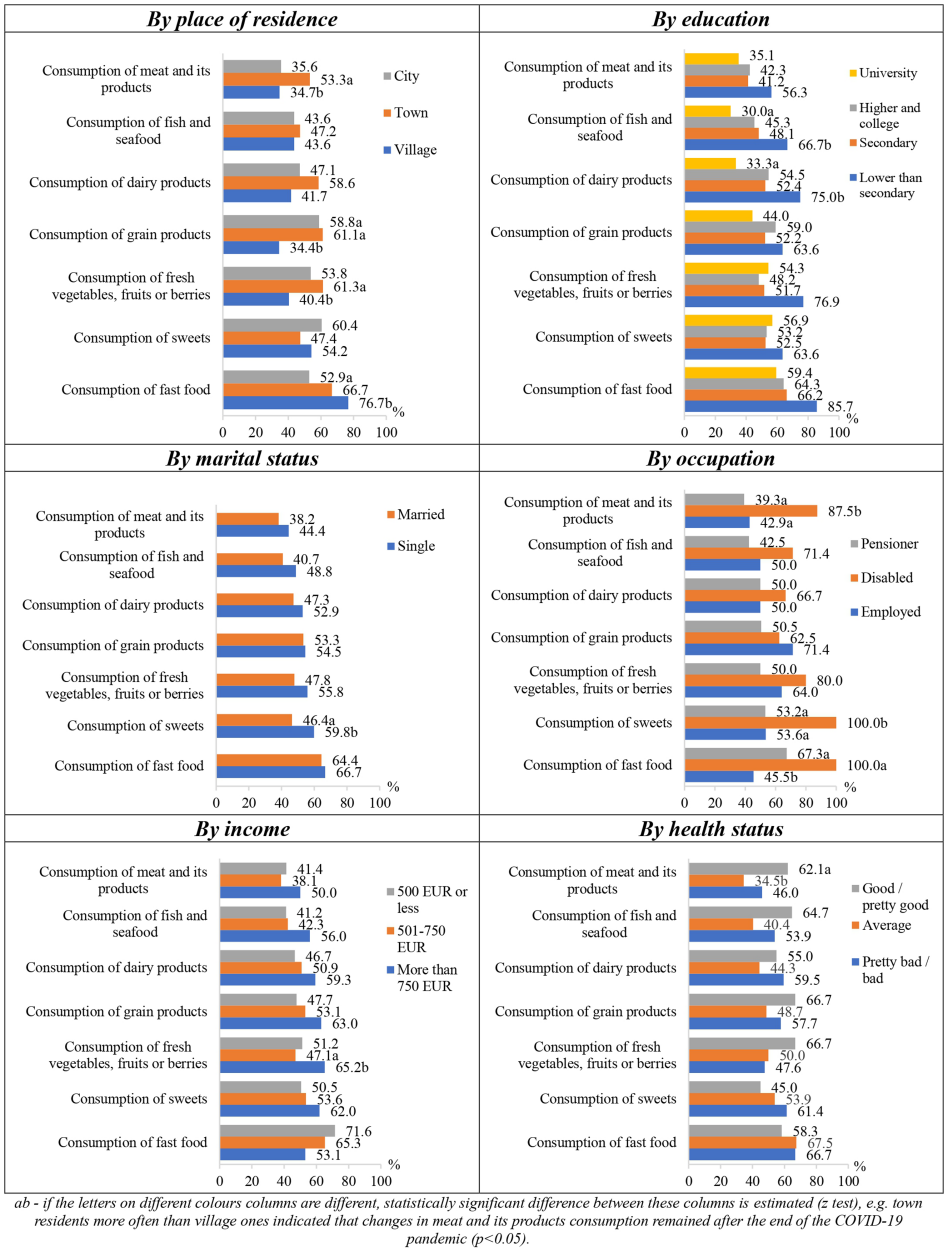
Changes in eating habits that remained after the end of the COVID-19 pandemic depending on socio-demographic factors.

Changes in eating habits after the pandemic were statistically significantly (*p* < 0.05) dependent on the sociodemographic characteristics (Figure 6). The tendencies were observed that the eating habits formed during the COVID-19 pandemic mostly remained in smaller cities, in groups of people with lower than secondary education, the singles, disabled persons and people with income above 750 EUR.

**Figure 7:**
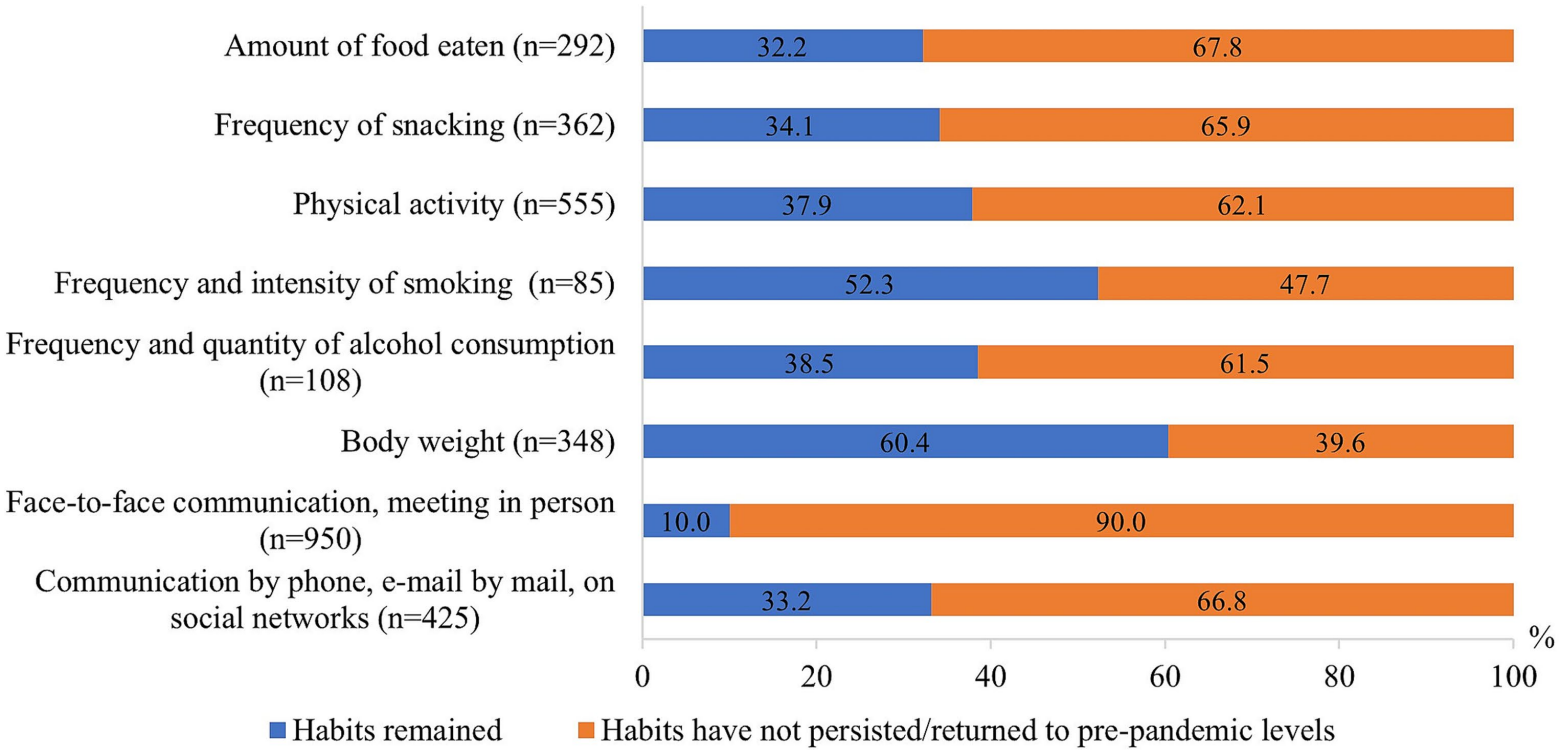
Changes of other lifestyle factors after the COVID-19 pandemic.

When evaluating the changes in other lifestyle factors after the end of the COVID-19 pandemic, it was observed that changes in body weight (60.4%) as well as smoking frequency and intensity (52.3%) that occurred during the pandemic mostly remained. After the end of the pandemic, communication by “live” meeting (90.0%) and communication by phone, e-mail or on social networks (66.8%), as well as the amount of food eaten (67.8%), frequency of snacking (65.9%), physical activity (62.1%) and frequency of alcohol consumption and quantity (61.5%) mostly returned to the pre-pandemic level (Figure 7).

**Figure 8:**
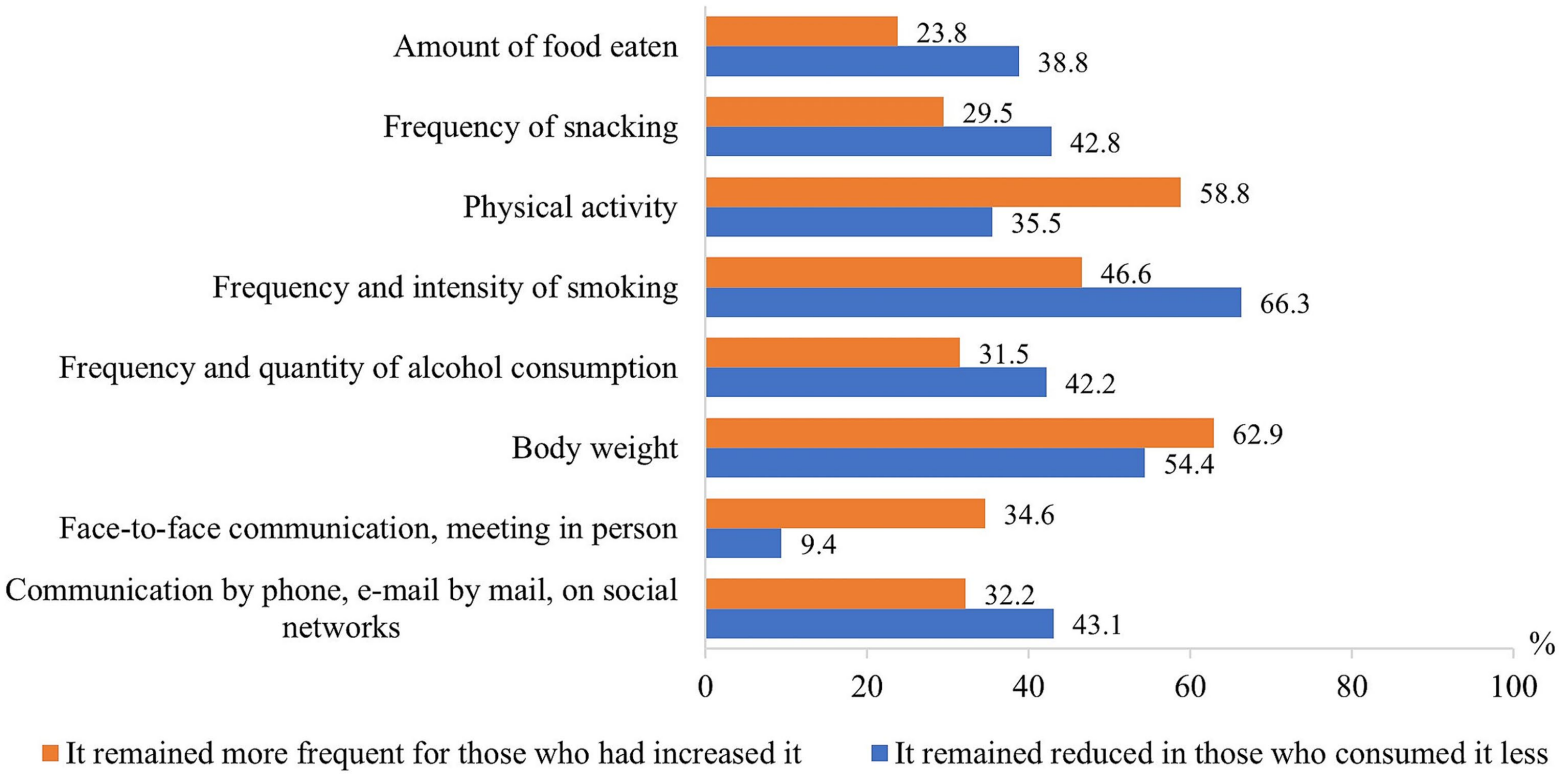
Proportion of respondents who maintained changes formed during the pandemic in other lifestyle factors after the end of the COVID-19 pandemic.

Physical activity, body weight, and face-to-face communication were more likely to be maintained after the pandemic in those who reported it increased during the pandemic, while the amount of food eaten, frequency of snacking, smoking and alcohol consumption, and communication with relatives remotely were more likely to be maintained in those who reported it decreased during the pandemic (Figure 8).

**Figure 9:**
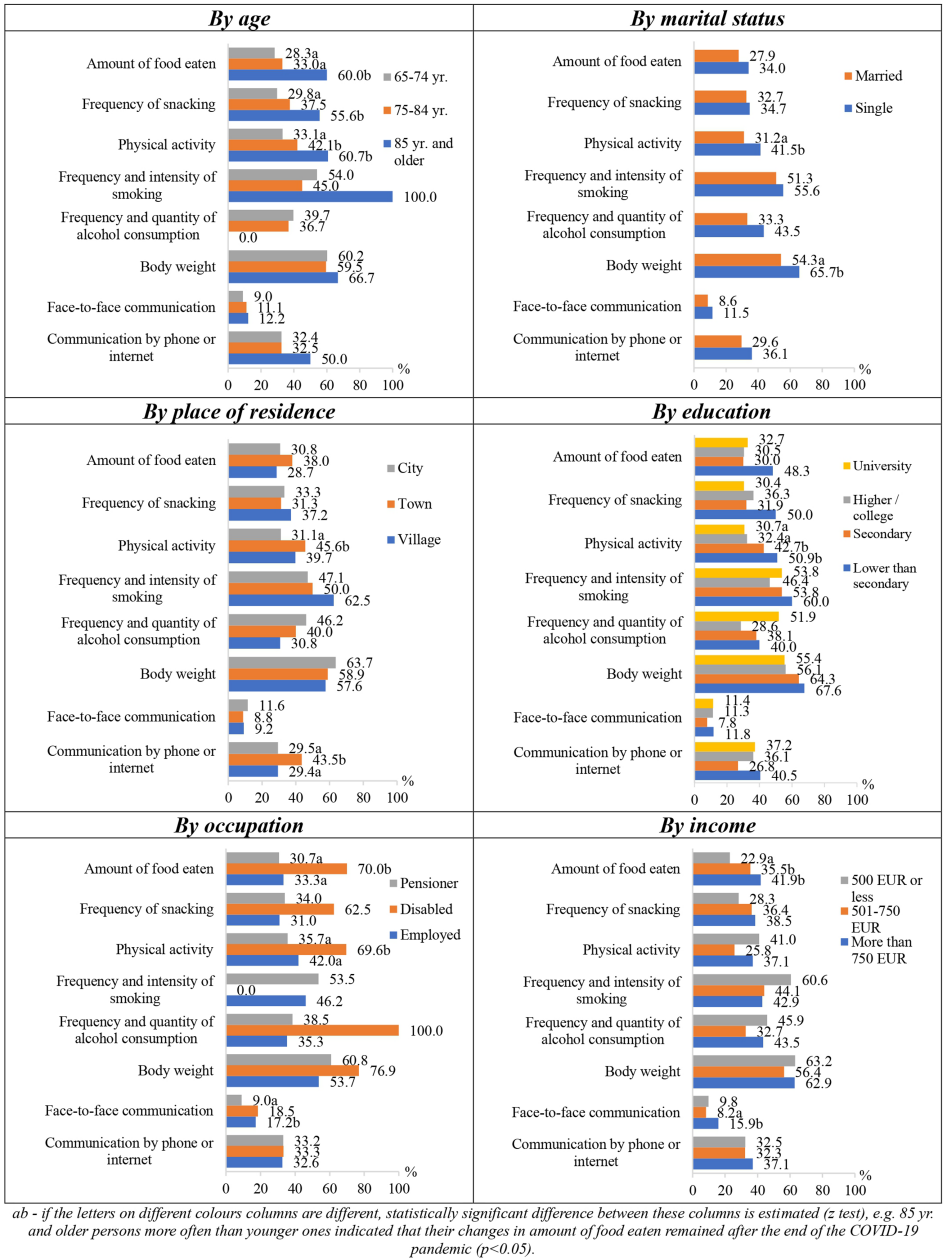
Changes in other lifestyle factors that remained after the end of the COVID-19 pandemic depending on socio-demographic factors.

Changes in lifestyle factors after the COVID-19 pandemic depended statistically significantly (*p* < 0.05) on various sociodemographic factors (Figure 9). The lifestyle habits formed during the pandemic mostly remained in groups of older, having less than secondary education, single, and disabled persons. Residents of smaller cities statistically significantly more often indicated that the changes in physical activity remained after the pandemic, compared to those living in big cities, as well as the residents of smaller cities differed from other groups in stating that the habits of communication formed over a distance remained. People with an income of more than 500 EUR more often reported that the amount of food eaten remained unchanged.

Obese people, as compared to overweight or low/normal weight ones, more often indicated that changes in their physical activity (48.6% vs. 34.6% vs. 34.0%) and body weight (71.1% vs. 54.7% vs. 56.9%) remained after the pandemic. Also, the respondents, who rated their health as pretty poor/poor, more often indicated that the lifestyle habits formed during the pandemic remained in the post-pandemic period.

## 4 Discussion

Coronavirus was a major health threat for older adults during the period of pandemics. It is well reported that mortality from COVID-19 was strongly associated with the high prevalence of chronic non-communicable diseases among the older adults (13). Therefore, lifestyle factors contributing to the development of chronic non-communicable diseases must be seriously considered in this age group of population.

A healthy diet is an important health-enhancing factor. A balanced diet strengthens the immune system both through the consumption of anti-inflammatory nutrients and the consumption of fermented foods by the gut microbiota, which provide metabolic compounds involved in the homeostasis of the inflammatory process. Therefore, it is very important to understand whether the period of COVID-19 quarantines may have affected the quality of nutrition and to what extent such changes may have had a long-term effect on the health of the population. An unhealthy diet combined with a concomitant decrease in physical activity can negatively affect health status, leading to an increased prevalence of obesity and other risk factors, which in turn can increase the risk of complications from COVID-19. Fifty-nine studies analyzed changes in eating habits as reflected by the amount of food eaten, cooking at home, snacking, and consumption of takeaway or home delivery. Most of the survey respondents indicated that they increased the number of meals per day and the amount of food eaten during the COVID-19 quarantine. In addition, during the quarantine, cooking at home increased, home-cooked food was eaten more often, and at the same time eating out decreased. Analysis by geographic region revealed substantial similarities in changes in eating patterns across the world, although increases in daily meals were less pronounced in Asian studies than in other regions (14). During a cross-sectional survey conducted in the United Kingdom, 79% of the participants suggested that at least one of the five lifestyle factors studied (healthy diet, overeating, exercise, sleep, alcohol consumption) had worsened. Subjects diagnosed with mental illness or obesity were particularly at increased risk of weight gain during the COVID-19 crisis (15). A similar study in Spain found an increase in emotional eating, “cravings” (the desire to consume certain foods) and eating to compensate for boredom or anxiety after weight gain (16).

Among the sudden changes in public life associated with the impact of the COVID-19 pandemic, a significant decrease in physical activity and a prolonged sedentary lifestyle can be observed. Restrictions on physical activity resulted from closed sports centers and limited social mobility. Social distancing and telecommuting may have contributed to sedentary lifestyles and increased sitting time during the day, as well as shorter periods of vigorous physical activity during leisure time and reduced physical activity, adverse changes in motivation, and individual perceptions of fatigue (17). A population-based survey, conducted in suburban Japan, revealed that due to the COVID-19 pandemic regular/daily exercise decreased – it was reported by the 30% of study participants. The health-related quality of life of these older adults deteriorated independently of their age, gender, body mass index, baseline status of activities of daily living, and musculoskeletal symptoms (18). Many studies have shown that regular physical activity helps prevent the most common chronic diseases, such as diabetes, hypertension, cardiovascular disease, cancer, chronic kidney disease, obesity, osteoarthritis, etc. (19). There is evidence that older adults who engaged in regular physical activity during COVID-19 quarantines had higher stress resilience scores and less symptoms of depression and anxiety (20). The findings from other countries are somewhat like the Lithuanian, nevertheless our study disclosed some specific peculiarities, particularly related to sociodemographic inequalities among older adults of the country.

### 4.1 Limitations of the study

We collected data about the lifestyle factors among the older adults during and after the COVID-19 pandemic. One of the potential limitations of the current study could be related to the challenges in remembering precisely about their lifestyle factors during the COVID-19 pandemic, because it happened a few years ago. However, this type of limitation could be seen in many survey-based studies where past events are examined.

### 4.2 Advantages of the study

The important advantage of this study is that it is population-based survey, involving a representative sample of Lithuanian population over 65 years old, therefore the conclusions can be generalized to the entire older adults population of Lithuania. Also, it is important to note, that it examines not only lifestyle factors during the COVID-19 pandemic, but also after the pandemic, which helps to evaluate lifestyle changes related to the COVID-19 pandemic and look at the perspectives in lifestyle of the older adults.

## 5 Conclusion

Most of the eating habits, e.g., the amount of food eaten, the frequency of snacking, and the body weight of the older adults did not change during the COVID-19 pandemic in Lithuania. Respondents noted that their physical activity decreased, face-to-face communication with relatives became rarer, while communication by phone, Internet or social networks increased. During COVID-19 pandemic, the increased frequency of snacking was more often indicated by the persons with lower than secondary education, working people, and those receiving higher income. An increased snacking and body weight were more prevalent among younger, single or overweight persons; increased weight during the pandemic was also more often mentioned by persons who assessed their health poorly. Physical activity decreased more often among rural residents and groups with higher or college education; face-to-face communication has become rarer among younger people, rural residents, pensioners, the disabled, people with an income of more than 750 EUR and those who are overweight. Remote communication has increased among women, residents with a university education and people with an income of 501–750 EUR (*p* < 0.05).

Nearly half of the respondents indicated that the changes in food consumption, smoking frequency and intensity that occurred during the COVID-19 pandemic remained after the pandemic, while the other half indicated that they returned to the pre-pandemic level. After the pandemic, the habits of consuming fast food, confectionery or other sweets, as well as the changes in body weight mostly remained, while the characteristics of communication with the close relatives, friends and family members mostly returned to the pre-pandemic level. Most of the healthy lifestyle habits, formed during the pandemic, persisted after it ended. The lifestyle habits, formed during the COVID-19 pandemic, more often remained in the groups of older persons, residents of smaller cities, those with lower than secondary education, higher income, single, disabled, obese people, and those with poorly self-perceived health (*p* < 0.05).

The current study adds to the research, confirming the impact of pandemics on the lifestyle of older adults, disclosing sociodemographic and spatial inequalities. Identification of the vulnerable groups of older adults is essential for further prediction and development of preventive measures for the future, to improve preparedness for the possible health disasters.

This study is considered as having high practical significance, since the most vulnerable groups among older adults of Lithuania have been detected in terms of lifestyles. Health care providers should pay particular attention to those living in rural areas, the disabled, single persons, as well as those with already prevalent lifestyle risk factors, such as overweight and obesity. Disease prevention and health promotion programs, delivered by primary health care centers and municipality public health bureaus are the key players in reducing inequalities in health care and improving health for older adults of Lithuania.

## Data Availability

The raw data supporting the conclusions of this article will be made available by the authors, without undue reservation.
